# Biological response of chemically treated surface of the ultrafine-grained Ti–6Al–7Nb alloy for biomedical applications

**DOI:** 10.2147/IJN.S197099

**Published:** 2019-03-06

**Authors:** Diego Pedreira de Oliveira, Tatiane Venturott Toniato, Ritchelli Ricci, Fernanda Roberta Marciano, Egor Prokofiev, Ruslan Z Valiev, Anderson Oliveira Lobo, Alberto Moreira Jorge Júnior

**Affiliations:** 1 Department of Materials Engineering, Federal University of São Carlos, São Carlos 13565-905, São Paulo, Brazil, moreira@ufscar.br; 2 Institute of Research and Development, University of Vale do Paraíba, São Paulo 12244-000, Brazil; 3 Scientifical and Technological Institute, Brasil University, São Paulo 08230-030, Brazil; 4 Saint Petersburg State University, Saint Petersburg 199034, Russia; 5 Institute of Physics of Advanced Materials, Ufa State Aviation Technical University, Ufa 450000, Russia; 6 LIMAV - Interdisciplinary Laboratory for Advanced Materials, Department of Materials Engineering, UFPI - Federal University of Piauí, Teresina 64049-550, Piauí, Brazil, lobo@ufpi.edu.br; 7 University of Grenoble Alpes, CNRS, Grenoble INP-LEPMI, and SIMAP Labs, Grenoble 38000, France, moreira@ufscar.br

**Keywords:** SPD, ECAP, UFG Ti–6Al–7Nb alloy, implants, surface treatment, biological response

## Abstract

**Background:**

Nanophase surface properties of titanium alloys must be obtained for a suitable biological performance, particularly to facilitate cell adhesion and bone tissue formation. Obtaining a bulk nanostructured material using severe plastic deformation is an ideal processing route to improve the mechanical performance of titanium alloys. By decreasing the grain size of a metallic material, a superior strength improvement can be obtained, while surface modification of a nanostructured surface can produce an attractive topography able to induce biological responses in osteoblastic cells.

**Methods:**

Aiming to achieve such an excellent synergetic performance, a processing route, which included equal channel angular pressing (ECAP), hot and cold extrusion, and heat treatments, was used to produce a nanometric and ultrafine-grained (UFG) microstructure in the Ti-6Al-7Nb alloy (around of 200 nm). Additionally, UFG samples were surface-modified with acid etching (UFG-A) to produce a uniform micron and submicron porosity on the surface. Subsequently, alkaline treatment (UFG-AA) produced a sponge-like nanotopographic substrate able to modulate cellular interactions.

**Results:**

After several kinds of biological tests for both treatment conditions (UFG-A and UFG-AA), the main results have shown that there was no cytotoxicity, expressed alkaline phosphatase activity and total protein amounts without statistical differences compared to control. However, the UFG-AA samples presented an attractive effect on the cell membranes, and cell adhesions were preferentially induced as compared with UFG-A. Both conditions demonstrated cell projections, but for UFG-AA, cells were more widely dispersed, and more quantities of filopodia formation could be observed.

**Conclusion:**

Herein, the reasons for such behaviors are discussed, and further results are presented in addition to those mentioned above.

## Introduction

Many strategies have been employed to improve cellular attachment and consequent tissue growth on the surface of nanophase/grain titanium alloys for biomedical applications. Within the last ten years, nanophase/grains have been considered as an essential and emergent strategy to modify titanium alloys, specifically for bone tissue engineering. Indeed, bulk and surface properties have a substantial influence on implant performance, and both can be altered to achieve suitable performance for many biomaterials. Regarding titanium and titanium alloys, static and dynamic mechanical behavior can be modified by enhancing the bulk nanostructure of metallic materials.^1^,^2^ Mechanical stability is imperative to produce reliable medical implants, decreasing the incidence of failures and consequently, revision surgeries for these implants; moreover, smaller-sized implants can be produced to supply the same functionality with less invasive surgeries necessary to perform the implantation.^3^

The outer surface region that is in contact with biofluid, ions, molecules, macromolecules, and cells plays a vital role in the biological stability of implantable biomaterials, which is equally important as the mechanical stability.

Macroscale-designed nano/micro-roughness can improve primary mechanical stability,^4^ and micro- and nanosurface features are crucial for driving cell events, from adhesion to differentiation,^5^ including osteoblastic lineage and mineralization, both of which are necessary to acquire the biological stability of the implants.^6^ In this context, providing both macro and nanoscale properties, an overall improvement can be expected. Bulk nanostructured metals obtained after performing severe plastic deformation (SPD) is a highly suitable processing route that improves the performance of conventional titanium materials for implants.^7^–^9^ By decreasing the average grain size of bulk metallic materials through such processing, outstanding strength can be acquired. Additionally, surface modifications of nanostructured metals can provide topographic features that can induce biological responses from bone-related cells when interacting with the metallic substrate.^10^,^11^ Indeed, in the literature, SPD has been widely described as an important metallurgical processing route to improve the mechanical performance of metals and alloys.^12^–^15^ A well-designed sequence of hot, warm, and cold metallurgical deformation can be employed to acquire tailored bulk nano-structures and optimize mechanical responses for implant material performance.^13^,^16^

SPD technique using equal channel angular pressing (ECAP) has been used to create ultrafine grain sizes in many metals and alloys.^1^,^17^ A successful route, which includes heat treatment (water quenching and overaging), a sequence of ECAP passes, followed by a sequence of hot and cold extrusion and stress relief by annealing at low temperatures, has been developed for the production of ultrafine-grained (UFG) Ti–6Al–7Nb alloy.^18^ This procedure was capable of producing rods with an average grain/subgrain size of about 200 nm, and an ultimate tensile strength of about 1,470 MPa, which may also improve wear resistance.

Treatments on surfaces of Ti and Ti alloys have also been reported in the literature.^19^–^22^ Notably, one useful and straightforward way for modifying surfaces was performed by using an acid medium to produce a uniform micron, and submicron porosity on the surface and, subsequently, an alkali treatment was performed to produce either a sponge or coral-like nanotopographic substrate enabling modulation of cellular interactions.^19^

The work herein aimed to establish a synergistic processing method for Ti alloys, combining SPD processing followed by surface modification, with the expectancy of improved mechanical properties of the material and biological responses. Such a methodology would ultimately induce osseointegration paths for the Ti–6Al–7Nb alloy into the bone. For such objectives, UFG microstructure was produced in bars of the alloy, while surface modification treatments were performed for micro–nano topographic alteration. Biological responses for both UFG acid-treated (UFG-A) samples and UFG acid- and alkaline-treated (UFG-AA) samples were obtained for MG63 osteoblastic-like cells.

## Materials and methods

### Material and processing

The chemical composition of Ti–6Al–7Nb (ASTM F1295) alloy by weight was: Ti-base, Al – 6.17%, Nb – 7.05%, Fe – 0.14%, O – 0.17%, C – 0.01%, and N – 0.03%. Hot-rolled bars of the Ti–6Al–7Nb alloy, with an initial average grain size of around 10–15 µm, were used for combined SPD treatment. The processing route included heat treatment (water quenching and overaging), six passes of ECAP at 600°C followed by hot extrusion (four passes at 300°C) and cold extrusion, and, finally, annealing at 500°C for 1 hour. In this way, UFG rods with a diameter of 12 mm and a length of 50 mm were produced.^18^ Disks of 2-mm thickness were cross-sectioned from Ti–6Al–7Nb UFG rods for subsequent procedures, as described hereafter.

### Surface chemical treatments

Ti–6Al–7Nb disks were wet ground using 1500-grit SiC paper, sonicated in deionized water and air-dried.

Samples were chemically treated by immersion, first in a phosphoric acid solution for 30 minutes at 80°C and then immediately immersed in deionized water for 10 minutes. The obtained samples are referred to as UFG-A.

Subsequently, dried samples were alkaline treated in 10 mol/L NaOH solution at 60°C for 24 hours, hereafter referred to as UFG-AA samples.

### Characterization by electron microscopy

#### TEM characterization

The microstructures were analyzed by transmission electron microscopy (TEM, FEI-TECNAI G2 LaB_6_) coupled to an orientation-phase mapping precession unit NanoMEGAS ASTAR with a Digistar P1000 unit. This system can acquire thousands of electron diffraction spot patterns, offering the opportunity to perform automated crystal orientation mapping in micro- or nanoprobe mode. Such a mapping is analogous to using electron backscattered diffraction (EBSD) in scanning electron microscopy (SEM). However, its resolution can go down to 1 nm, allowing the observation of features in the nanometric range, and enabling the analysis of heavily deformed samples as for ECAP-processed samples. Hereafter, this system will be referred merely to as ASTAR.

For TEM analysis, sheets of materials were initially obtained by cutting UFG rods cross-sectionally, with a thickness of about 100 µm. Disks of 3 mm were then punched out from the sheets and thinned down to about 50 µm by conventional grinding. Finally, thin foils were prepared using ion milling in Gatan Precision Ion Polishing System (model 691) under conditions in which damage is avoided in the structure during thinning (2.5 keV at the initial stage of polishing and 1.6 keV at the final stage).

#### SEM characterization

Surface morphology of the samples after all stages of surface treatment and after bioactivity tests was characterized by SEM using an (FEI) Philips XL30-FEG equipped with an Oxford Link ISIS 300 and energy-dispersive X-ray spectroscopy (EDS) to analyze chemical alterations in microregions.

### Roughness and effective area measurements

Confocal laser scanning microscopy (CLSM), OLYMPUS LEXT OLS 4000 was used to analyze surface roughness parameters taken from 3D images: amplitude (S_a_, S_z_), spatial (S_sk_ and S_ku_), and hybrid parameters (S_dr_).

### Wettability and cell adhesion forces

The contact angle (CA) measurements were performed using Sessile Drop Technique via Easy Drop DSA 100S Krüss, dripping around 2 µL of deionized water (polar), and diiodomethane (dispersive) components in an atmosphere where temperature and pressure were controlled. Measurements were performed immediately after dripping, thus avoiding evaporation and other perturbations. Surface energy for each sample was calculated by thermodynamic principles using the methodology proposed by Owens and Wendt.^23^

### Bioactivity assay in Simulated body fluid (SBF) medium

SBF solution was used to analyze the in vitro bioactivity. The method of Kokubo and Takadama^24^ was used to prepare the SBF solution. Briefly, the solution was prepared using NaCl, KCl, K_2_HPO_4_, CaCl_2_⋅2H_2_O, MgCl_2_⋅6H_2_O, NaHCO_3_, and Na_2_SO_4_. All reagents were dissolved in deionized water (the full procedure is described in Oliveira et al^20^).The pH of the solution was adjusted to 7.4. Then, the analyzed samples were incubated with 20 mL of SBF (polyethylene tubes) and stored for 14 days under constant agitation of 75 rpm in a temperature-controlled chamber at 36.5°C. After such an incubation time, samples were gently washed with deionized water and then dried at room temperature.

### Cell culture

All in vitro tests were performed using MG63 human osteoblast-like cells (ATCCR^®^ CRL-1427™). The experiments were carried out in triplicate. DMEM (Thermo Fisher Scientific, Waltham, MA, USA) supplemented with FBS (Thermo Fisher Scientific), streptomycin (Thermo Fisher Scientific), and penicillin (Thermo Fisher Scientific) were used as cell culture media. The following environmental conditions was used: 37°C in a humidified atmosphere of 5% (v/v) CO_2_. Previously, UFG alloys were sterilized in absolute ethanol. As a control, cells were grown onto a culture plate for all in vitro analyses.

### Cell viability

For viability tests, 1×10^5^ cells/well osteoblast cells were inserted individually in 24-well plates for 24 hours. After the culture period, cells were trypsinized with Trypsin–EDTA 0.25% (Thermo Scientific). Next, the cell suspension was stained using Trypan Blue solution 0.4% (Sigma Aldrich) and counted (Countess – Invitrogen).

### Total protein measurement

Total protein content was determined following the Lowry method for protein quantitation using a cell concentration of 2×10^4^ cells/well for 14 days. All procedures were performed according to those suggested by the method of Andrade et al.^25^ To analyze the protein expression, the absorbance was measured after 14 days using a spectrometer (680 nm, EL808IU, Biotek Instruments, Winooski, USA). The total protein content was expressed in µm/mL.

### ALP activity

ALP activity was carried out after 14 days. The experimental procedure was the same reported in Rodrigues et al.^26^ The proposed manufacturer’s procedure protocol was used (LabstestDignostica, Belo Horizonte, BR). The ALP assay was realized in triplicate. Briefly, the sterilized samples were put into 24-well plates, and cells were seeded on samples and culture media was added at each well (1 mL). After 14 days, the culture media was removed, the cells were lysed, and transferred to 96-well plates. The absorbances were measured (Shimadzu Europa GmbH UV 1203) at 590 nm and the total ALP activity was normalized by total protein level and the value expressed in thymolphthalein/h/mg protein/mL.

### Cell adhesion and morphology by SEM analysis

SEM (EVO MA10, Zeiss) characterized the morphology of osteoblasts (2×10^4^ cells/mL) grown on the titanium alloy samples after 25 minutes and 24 hours. The number of attached cells after each time was analyzed using ImageJ. After each time, the samples containing attached cells were fixed using 4% paraformaldehyde/2.5% glutaraldehyde in PBS for 10 minutes. After that, the samples were fixed using graded ethanol solution (50%, 70%, 90% and 100%, 10 minutes each). Next, the samples were fixed using ethanol:hexamethyldisilazane solution (1:1) for 30 minutes. Finally, the samples were fixed in hexamethyldisilazane for 30 minutes (room temperature). Before SEM analysis, the samples with fixed cells were sputter-coated with a thin gold film. Cell area monolayer percentage covering the sample surface was quantified with the ImageJ software from SEM images at 1,000× magnification.

### Statistical analyses

The data were analyzed and only a normal distribution was indexed (ANOVA, post-hoc Tukey’s test and level of significance at a 95% CI, *P*<0.05). All in vitro assay results were expressed as the mean ± SDs. Kolmogorov–Smirnov test was performed to analyze the statistical significance.

## Results

### Bulk characterization by TEM

**Figure 1:**
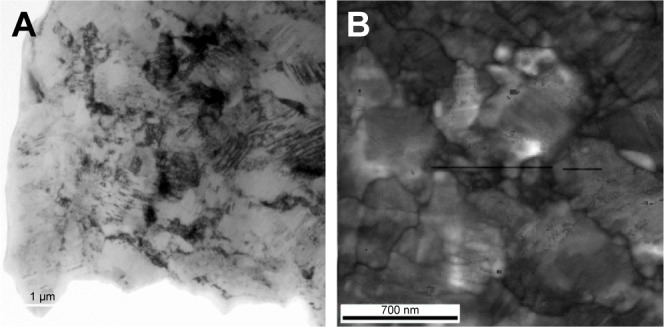
(**A**) BF-TEM image of the UFG Ti–6Al–7Nb. (**B**) VBF-TEM image obtained from the ASTAR system of a region of (**A**). **Abbreviations:** BF, bright field; TEM, transmission electron microscopy; UFG, ultrafine-grained; VBF, virtual bright field.

Figure 1A presents a bright field (BF) TEM micrograph taken from a region of the cross-section of the UFG Ti–6Al–7Nb rod. Figure 1B displays a virtual bright field (VBF) TEM image from an area of Figure 1A. The VBF is a reconstructed image produced by the ASTAR system using the information of brightness and contrast present in the transmitted beam of the diffraction pattern. Both figures confirm grain sizes in the bulk material ranging between the nano- and submicron scale. However, it is also possible to observe that the sample is still severely deformed even after the last step of the processing route (annealing at 500°C for 1 hour).

**Figure 2:**
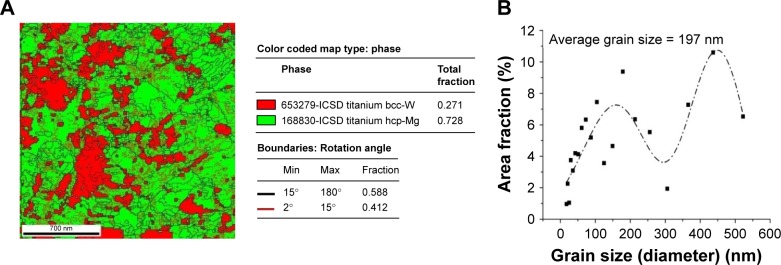
(**A**) Phase mapping superimposed with the map of boundaries, showing that most of the UFG Ti–6Al–7Nb sample is composed by α-Ti-(hcp) phase (73%) where the blue lines represent high-angle boundaries or grain boundaries, (θ>15°), and the red lines represent low-angle boundaries or subgrain boundaries (2°<θ<15°). (**B**) Graph of the grain size distribution. **Abbreviation:** UFG, ultrafine-grained.

Figure 2A presents a map of boundaries superimposed on the VBF image of Figure 1B. In this picture, fractions of high- and low-angle (θ>15° blue lines and 2°<θ<15° red lines, respectively) boundaries are an indirect way of measuring the recrystallized volume fraction. This map revealed that around 60% of boundaries were those with a high-angle, indicating partial recrystallization after the last step of the processing route and confirming that the sample is still deformed. The graph of grain size distribution presented in Figure 2B indicates a bimodal behavior with grain sizes ranging from a few tens of nanometers to about 550 nm, having an average size of approximately 197 nm, also confirming the observations in Figure 1A and B.

The phase mapping superimposed on the map of boundaries obtained using the ASTAR system, collected from the same region of Figure 1B is presented in Figure 2. By indexing this colored map (right side of the figure), most of the sample is notably formed by the α-phase (hcp-Ti) expressed by the green color, which constitutes about 73% of the sample. The other indexed phase was the β phase (bcc-Ti) denoted by the red color, corresponding to about 27% of the sample.

### Surface characterizations

**Figure 3:**
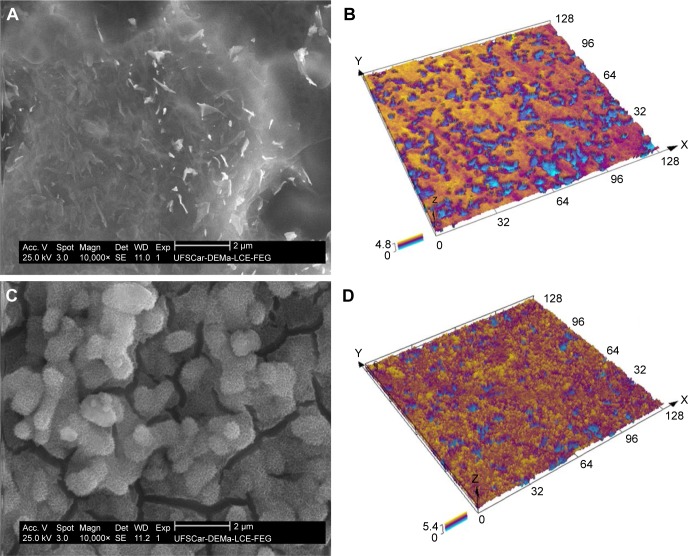
SEM images showing the surface morphology (**A** and **C**) and 3D virtual surface area (**B** and **D**) after chemical treatment on the surface of UFG Ti–6Al–7Nb alloy: (**A** and **B**) UFG-A and (**C** and **D**) UFG-AA. **Notes:** Additionally, quantitative chemical microanalysis (not shown) indicated a reduction of Nb from 7 wt% to about 4.5 wt%, meaning that possibly some β-phase has been removed by chemical etching. Scales are in µm. **Abbreviations:** SEM, scanning electron microscopy; UFG, ultrafine grained; UFG-A, acid treated; UFG-AA, acid and alkaline treated.

SEM analysis was performed to evaluate the influence of chemical treatments on surface morphologies. Figure 3A and C presents topographic images for the surface-modified UFG Ti–6Al–7Nb alloy. As stated in the methodology (section Surface chemical treatments), UFG-A denotes acidly etched, and UFG-AA denotes acid-etched plus alkali treatment, shown in Figure 3A and C, respectively. From these pictures, it is possible to observe that very tiny particles were formed on the surface of the UFG-A samples (Figure 3A), while the acid plus alkali treatment induced the formation of a nanotopographic coral- or sponge-like morphology (Figure 3C) on the surface of the UFG-AA samples.

CLSM was performed to assess virtual 3D topographic representative image for UFG-A (Figure 3B) and UFG-AA (Figure 3D) samples. Comparing the ASTAR phase mapping of Figure 2 to the CLSM images, it is possible to infer that the β-phase has been removed by chemical etching. This conclusion is because there are regions where the porosity was more in-depth than in other areas (as observed in Figure 3B and D), suggesting that the acid medium preferentially subtracted such a phase. Importantly, this is corroborated by the results of chemical analysis mentioned above. Roughness parameters were measured from these 3D images. Table 1 provides the values for the following: amplitude (S_a_, S_z_), spatial (S_sk_ and S_ku_), and hybrid parameters (S_dr_). Surface average roughness (S_a_) is not able to characterize lateral roughness.^27^ Hence, additional parameters were included for this reason. Skewness can be observed using S_sk_ parameter and the distribution of surface features can be observed by kurtosis parameters (S_ku_).

**Figure 4:**
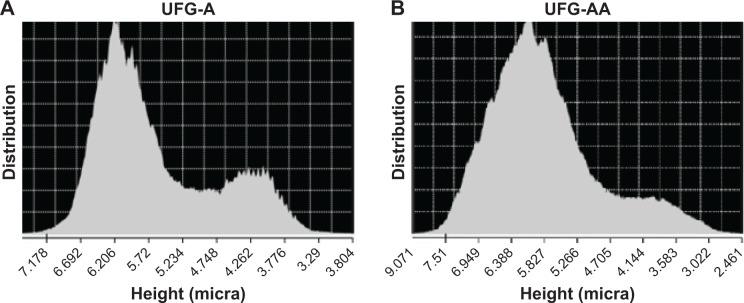
Height distribution of surface characteristics obtained by CLSM: (**A**) UFG-A and (**B**) UFG-AA samples. **Abbreviations:** CLSM, confocal laser scanning microscopy; UFG, ultrafine grained; UFG-A, acid treated; UFG-AA, acid and alkaline treated.

Figure 4 presents the height distribution of the surface for each sample, UFG-A and UFG-AA, extracted from the CLSM analysis, in which the bimodal distributions in both sample groups can be observed. The surface of UFG-AA presents an enlargement of the normal distribution and a kurtosis higher than 3.0. Both surfaces exhibited a left-shifted, negatively skewed curve in comparison to the control. Despite having almost the same maximum position at around 6.1 µm, these results indicate that surfaces of UFG-A samples are smoother than those of UFG-AA samples.

### Wettability and cell adhesion forces

Table 2 shows the results of CA measurements. These results indicate that the UFG-AA samples presented highly hydrophilic responses in comparison with those of UFG-A samples. The analysis of the dispersion behavior of a liquid droplet in contact with the surface of samples revealed that both conditions presented amphiphilic behavior.

Table 3 shows calculations of adhesion energy, whose values were based on the surface energy of MG63 human osteoblast cells. These results clearly show that the surface- free energy, as well as the interfacial free energy of adhesion, is much higher in UFG-AA samples compared to UFG-A samples.

### Bioactivity tests

**Figure 5:**
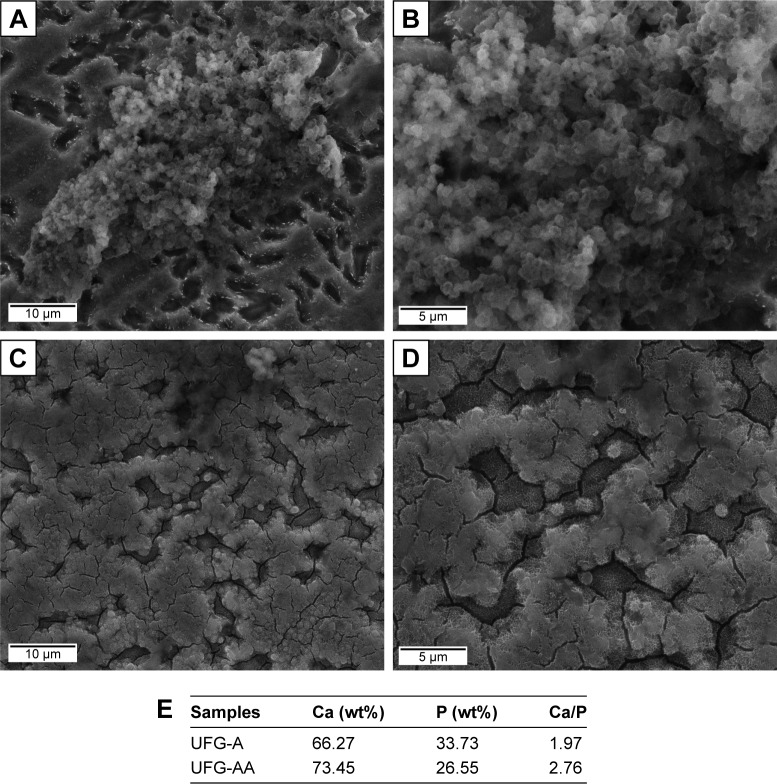
Surfaces of samples after immersion in SBF solution for 14 days: (**A**, **B**) for UFG-A samples and (**C**, **D**) for UFG-AA samples. (**E**) Ca and P deposition analyzed by EDS. **Abbreviations:** EDS, energy-dispersive X-ray spectroscopy; SBF, simulated body fluid; UFG, ultrafine grained; UFG-A, acid treated; UFG-AA, acid and alkaline treated.

Figure 5 shows typical SEM micrographs for bioactivity results obtained for samples (UFG-A and UFG-AA) after 14 days of immersion in SBF solution and a table indicating the presence of Ca and P analyzed by EDS. Briefly, both samples presented bioactivity, and a globular apatite layer was obtained for UFG-A (Figure 5A and B) and UFG-AA (Figure 5C and D). The globular apatite deposition for UFG-A (Figure 5A and B) was more heterogeneous than those collected from UFG-AA (Figure 5C and D), which can be noticed by the deposition of Ca and P analyzed by EDS (Figure 5E). However, despite similarities, the UFG-AA (Ca/P ratio around of 1.96) samples showed a slightly lower deposition of Ca and P compared to UFG-A (Ca/P ratio around of 1.84), indicating that the UFG-A samples, with regions full of aggregate particles (Figure 5E), are slightly more bioactive than UFG-AA.

### Biological assay

**Figure 6:**
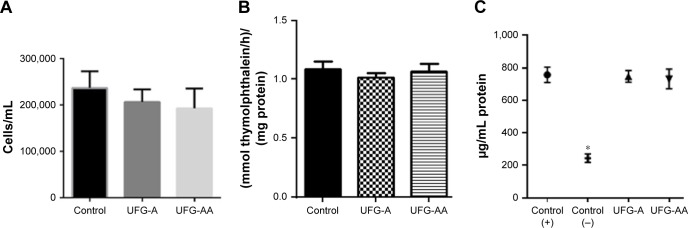
(**A**) Cell viability test using a trypan blue assay. (**B**) Graph showing results of ALP activity tests. (**C**) Total protein analysis. All biological assays were performed in triplicate and *P*<0.05 was considered statistically different. Only cells were used as a control. (**C**) A latex glove was used as negative control and only cells as positive control. *P<0.001. **Abbreviations:** UFG, ultrafine grained; UFG-A, acid treated; UFG-AA, acid and alkaline treated.

Figure 6 summarizes the performed biological assays. Figure 6A illustrates the cytotoxic effects on the cells after 24 hours. Such results indicate that the cytotoxicity of the reference sample is slightly higher than that of UFG-A and UFG-AA samples, for which no differences in cell viability were observed when compared to each other after cell culture. Figure 6B shows the ALP activity tests after 14 days. The results indicate that there are no significant differences between the sample groups UFG-A, UFG-AA, and the control. All conditions, including the reference sample, presented elevated ALP production. Figure 6C shows the results of total protein measurements. Clearly, there are no significant differences between the treated samples and the positive control, which all induced a high level of protein production in the cells compared to the negative control.

**Figure 7:**
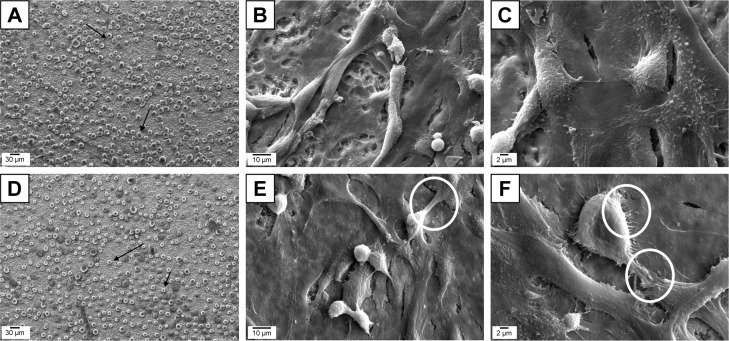
Representative SEM images of cell adhesion tests in cell culture. **Notes:** (**A**) UFG-A and (**D**) UFG-AA after 25 minutes, respectively. (**B** and **C**) UFG-A and (**E** and **F**) UFG-AA after 24 hours, respectively. The circles in images (**E** and **F**) illustrate filopodia formation on UFG-AA samples. **Abbreviations:** SEM, scanning electron microscopy; UFG, ultrafine grained; UFG-A, acid treated; UFG-AA, acid and alkaline treated.

Figure 7A and D shows the cellular adhesion onto UFG-A and UFG-AA, respectively, after 25 minutes of analysis (cells identified by arrows). Similarly, Figure 7B and C shows cellular adhesion cultivated onto UFG-A, and Figure 7E and F onto UFG-AA after 24 hours, respectively. There are differences in cell morphologies depending on the employed surface treatment. The circles in Figure 7E and F show filopodia formation on UFG-AA samples.

**Figure 8:**
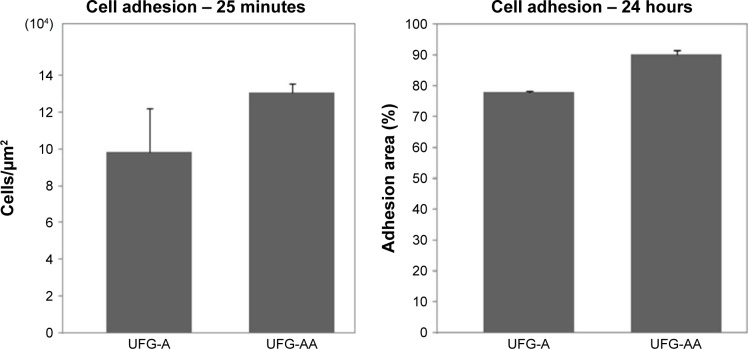
Graphs presenting the results of area fraction quantification of cell adhesion on UFG-A and UFG-AA samples after 25 minutes and 24 hours in cells’ culture. **Abbreviations:** UFG, ultrafine grained; UFG-A, acid treated; UFG-AA, acid and alkaline treated.

It was possible to quantify the area fraction of sample surface covered by the cell monolayer by calculating the area of the cells adhered to the surface divided by the total surface area, and the results are presented in Figure 8. After 24 hours of cell culture, UFG-A samples had an average of 78.2%±0.6% cell coverage of the surface, while UFG-AA samples had an average of 90.4%±1.1% cell coverage (statistical significance *P*<0.05).

## Discussion

Our experiments aimed to demonstrate the influence of surface modifications on high-strength UFG titanium alloy (Ti–6Al–7Nb) obtained by processing route which included ECAP, extrusion, and annealing.

According to TEM analysis, an efficient grain refinement was obtained on Ti–6Al–7Nb rods which were previously biphasic with an initial average grain size around 10–15 µm. After SPD, thermo-mechanical processing, and heat treatment a bimodal grain distribution (about 150 and 400 nm) was found with a final average grain size of approximately 200 nm (Figures 1 and 2). BF-TEM imaging (Figure 1A) and maps of boundaries obtained with the ASTAR system (Figures 2 and 3) revealed that samples were still deformed even after heat treatment, and that about 60% of the bulk material was recrystallized. Furthermore, phase mapping (Figure 3) showed an increased amount of α-phase (hcp-Ti) after processing. The same result was found by other authors,^12^,^13^ who observed a similar α+β microstructure in conventional alloys.

Additional surface chemical treatments induced the formation of different morphologies on sample surfaces. The acid treatment (UFG-A samples) produced very tiny particles on the sample surfaces (Figure 4A), while the acid plus alkali treatment (UFG-AA samples) induced the formation of a nanotopographic coral- or sponge-like morphology (Figure 4B). Both of them presented a bimodal distribution of surface heights in microscale analysis (Figure 5). However, despite a predominant normal distribution for both cases, UFG-AA samples were rougher than those of UFG-A (Figure 4 and Table 1). According to EDS chemical composition quantifications, decreased niobium content was observed after chemical treatments. This finding was associated with the β-phase distribution observed in the phase mapping of Figure 3, suggesting the removal of β-phase by acid etching, thus resulting in a surface with regions where the porosity was more in-depth than other regions (Figure 4B and D). Also, the deposition of calcium and related phosphorous compounds could be observed after soaking in biofluid for 14 days, demonstrating the enhanced bioactivity afforded by these surface treatments.

According to the mechanical point of view, the improvement of mechanical strength after SPD processing is expected.^18^ However, surface imperfections can lead to an expected decrease in surface-related mechanical properties, such as fatigue resistance. Small changes in surface roughness, eg, R_z_ above 3.0 µm for Ti–6Al–4V is conventional grained, lead to a critical decreasing to fatigue strength.^28^ Equilibrium between both conditions, surface features to improve osseointegration and roughness amplitude to avoid mechanical-related failures, is essential to obtain well-designed material for the biomedical application, especially for orthopedics that requires higher loadings compared to dental implants. Based on these analyses, the increased bulk yield strength is pointed as an important strategy to improve dynamic and static mechanical properties, as well bioresponses.^8^

Cell viability is fundamentally essential to assess cell metabolism when in contact with the modified surface of samples. After analyzing the results presented in Figure 6A, there were no statistical differences among the analyzed groups (*P*<0.05). Importantly, no cytotoxic events were observed for any group. Additionally, ALP activity and total protein amount (Figure 6B) presented no significant differences between the treated groups and positive control. Total proteins were also quantified, and no difference was identified between analyzed groups (Figure 6C). However, all analyzed groups expressed more total protein than control (as expected). All analyzed groups presented bone-related mineralization events at the same level compared to control conditions.

Additionally, when evaluating surface performance, it is essential to analyze cell shapes, which herein were evaluated according to the dispersion morphology induced by micro and nanosurface features. UFG-AA samples of the Ti–6Al–7Nb alloy had an attractive effect on the cell membranes (flattening), and cell adhesions were preferentially induced (more details can be seen in Figures 7 and 8). Adhesive performance of surface can be primarily attributed to the chemical environment and surface energy (identified by CA and surface analysis, Tables 2 and 3). Hydrophilicity and phosphorous content are known to play a vital role in attracting cells to a surface, in addition to the nanotopographic formation, which provides desirable surface features for cell anchorage.^29^–^31^ (as identified by EDS analysis shown in Figure 5E and Table 2). Furthermore, integrin has been noted as significant (not quantified here), localized ligand molecule for cells, inducing the adhesion, dispersion, and cell-to-substrate interactions.^32^

As observed in Figures 7 and 8, cell coverage was predominant for cells cultured on Ti–6Al–7Nb UFG-AA surfaces. Cell adhesion and consequently projections are fundamental to influence nucleus deformation, and consequently, differentiation paths.^33^ Both samples presented cell projections; however, cell dispersion was greater for UFG-AA, leading to a higher generation of filopodia.^34^,^35^ Indeed, for the UFG-AA, the surface treatment produced some nanostructures protruding from the surface, which most likely afford cell anchorage, and promote cell dispersion and motility.^36^

When determining the hydrophilicity of both samples via liquid drop tests on the surfaces (Table 2), both conditions presented amphiphilic behavior, attracting both polar and dispersive media. Some similar materials demonstrated the same behavior.^37^,^38^ This versatile surface energy behavior also contributed to improved cell adhesion (Table 3).

Overall, the UFG-AA material presented nanostructures on the surface that were able to improve biological responses compared to conventional materials (cellular spreading showed in Figure 8). This behavior suggests that UFG alloys can be essential to develop nanostructured, chemically treated implantable devices for osseointegration; conventional alloys do not provide the same nanostructured surface, similar gene expression to an AA treatment was already observed.^19^,^20^

UFG-A and UFG-AA presented high cell adhesion in as quickly as 25 minutes after seeding and was maintained after 24 hours. Additionally, after 14 days, samples presented calcium phosphate deposition; this suggests the high bioactivity of the samples. Based on differentiation data, cyto-skeleton projections and cell membrane deformation such as lamellipodia, actin-mediated bundle, and filopodia formation were induced by substrate topography, chemistry, and surface energy. Several studies indicate that cell dispersion behavior, associated with nucleus deformation, plays a vital role in promoting cell mineralization, osteoconductivity, and even osteoinductivity.^39^,^40^ In any case, a more complete and detailed investigation of cell behavior is still needed to identify the significance of the surface modification and properties affecting each cell response. For example, mineralization assays that showcase gene expression can evaluate the efficacy of these surface modifications to induce the osseointegration process.^19^,^21^

Thermodynamically, when the adhesion force (ΔF_Adh_) is negative, osteoblast cells display favorable attachment and dispersion over the surface. On the other hand, positive values indicate that surfaces do not present high attractive behavior for cells and less dispersion behavior can be observed (Table 3). The results are in agreement with those found in the literature.^23^ Precisely, for UFG-AA, very negative surface energy was calculated. This negative value coupled with the height profile normal distribution for the surface microtopography and the nanotopographic surface coating lead to an increase in cell adhesion and dispersion.

## Summary and conclusion

In this work, rods of the Ti–6Al–7Nb alloy were subjected following a processing route that included ECAP, hot and cold extrusion, and heat treatments used to produce UFG microstructure. Additionally, UFG samples were surface-modified with acid etching (UFG-A), and subsequent alkaline treatment (UFG-AA) was used to modify the previously obtained surface morphology. After that, several microstructural, chemical, and biological tests were performed to characterize both sample types (UFG-A and UFG-AA). From the obtained results and correlated discussions, the following conclusions can be drawn:

- The thermomechanical processing route successfully produced a microstructure with grain sizes of about 200 nm.
- Surface modification by acid etching produced a uniform micron and submicron porosity on the surface. The subsequent alkaline treatment modified the previous surface, producing a nanotopographic coral- and sponge-like effect on the substrate.
- Samples did not present any cytotoxicity.
- ALP activity and total protein amounts displayed no significant differences between both conditions. However, the UFG-AA samples presented a more attractive effect on cell membranes, and cell adhesions were preferentially induced.
- Both kinds of surface treatments demonstrated cell projections, but for UFG-AA cells were more dispersed and a higher filopodia formation was observed.
- A synergism between nanostructuring and surface treatment was obtained, where a nanotopographic substrate was sufficient to improve the cell extension on the substrate.

## Figures and Tables

**Table 1:** Values of surface roughness parameters: amplitude (S_a_, S_z_), spatial (S_sk_ and S_ku_), and hybrid parameters (S_dr_)

Samples	S_a_ (μm±σ)	S_z_ (μm±σ)	S_sk_ (±σ)	S_ku_ (±σ)	S_dr_ ratio^a^ (±σ)
UFG-A	0.786±0.054	8.521±0.415	−0.854±0.057	2.543±0.134	2.304±0.040
UFG-AA	0.687±0.021	12.623±3.186	−0.793±0.145	3.180±0.374	3.730±0.204

**Note:**

a S_dr_: developed interfacial area.

**Abbreviations:** UFG, ultrafine grained; UFG-A, acid treated; UFG-AA, acid and alkaline treated.

**Table 2:** CA measurements for UFG-A and UFG-AA samples

Samples	CA (°)	
	Water	Diiodomethane
UFG-A	87.23±6.58	73.66±8.16
UFG-AA	23.06±3.04	23.73±6.63

**Abbreviations:** CA, contact angle; UFG, ultrafine grained; UFG-A, acid treated; UFG-AA, acid and alkaline treated.

**Table 3:** Results of calculations of the energy of adhesion

Samples	Surface-free energy (mN/m)			Interfacial free energy of adhesion (ΔF_adh_) (mJ/m^2^)
	Dispersive (ϒ^d^)	Polar (ϒ) p	Total	
UFG-A	16.31	8.26	24.57	3.6
UFG-AA	34.96	38.30	73.56	−35.3

**Abbreviations:** UFG, ultrafine grained; UFG-A, acid treated; UFG-AA, acid and alkaline treated.
